# Dedicated Automatic Recall Hepatocellular Cancer Surveillance Programme Demonstrates High Retention: A Population‐Based Cohort Study

**DOI:** 10.1111/liv.70020

**Published:** 2025-02-10

**Authors:** Mayur Brahmania, Stephen Congly, Yashasavi Sachar, Kelly W. Burak, Brendan Lethebe, Jessie Hart Szostakiwskyj, David Lautner, Alexandra Medellin, Deepak Bhayana, Jason Wong, Henry Nguyen, Matthew D. Sadler, Meredith Borman, Alexander I. Aspinall, Carla S. Coffin, Mark Swain, Abdel‐Aziz Shaheen

**Affiliations:** ^1^ Division of Gastroenterology and Hepatology, Department of Medicine Schulich School of Medicine London Ontario Canada; ^2^ O'Brien Institute of Public Health Schulich School of Medicine London Ontario Canada; ^3^ Division of Internal Medicine, Department of Medicine Schulich School of Medicine London Ontario Canada; ^4^ Department of Oncology Cumming School of Medicine Calgary Alberta Canada; ^5^ Clinical Research Unit Cumming School of Medicine, University of Calgary Calgary Alberta Canada; ^6^ Department of Radiology Cumming School of Medicine, University of Calgary Calgary Alberta Canada

**Keywords:** HCC, liver cancer, surveillance ultrasound

## Abstract

**Introduction:**

Patient, clinician, and system‐related barriers may affect adherence to hepatocellular carcinoma (HCC) surveillance programmes. The impact of a dedicated automated recall HCC surveillance programme on retention rates in patients eligible for screening is unknown. We aimed to describe and evaluate a large HCC surveillance programme in a publicly funded healthcare system.

**Methods:**

Data were collected from January 1, 2013, to December 31, 2022, from a retrospective cohort of subjects enrolled in a publicly funded automated recall semi‐annual surveillance programme as per the American Association for the Study of Liver Disease HCC guidance in the Calgary Health Zone (~1.6 million), Canada. Patients were excluded if there was incomplete data or did not meet indications for surveillance. Cox regression was used to identify predictors of non‐retention to surveillance.

**Results:**

A total of 7269 patients were included. The median was age 55.5 years (IQR: 45.5–63.8), 60% were male, 46% were of Asian descent, 51% had HBV infection, and 36% had cirrhosis (35% alcohol‐related). Median follow‐up was 4.9 years (IQR: 1.5–7.2). Overall, 52% (*n* = 3768) of patients were retained in the surveillance programme, while 8.3% (*n* = 603) left for potential medical reasons, and 40% (*n* = 2898) were lost in follow‐up. The median time in the programme for those lost in follow‐up was 0.81 years (IQR: 0.0–2.8) compared to 6.75 years if retained (IQR: 5.6–8.6; *p* < 0.001). In multivariable Cox regression analysis, HCV aetiology (HR 1.41; CI 1.23–1.62, *p* < 0.01), African ethnicity (HR 1.20, CI 1.02–1.42, *p* = 0.03), and cirrhosis (HR 1.16, CI 1.05–1.28, *p* < 0.01) increased risk of dropout. On interaction analysis, Hepatitis B amongst cirrhotic patients also increased risk of dropout (HR 1.48, CI 1.05–2.07, *p* = 0.02).

**Conclusion:**

A dedicated automated recall HCC surveillance programme has a high retention rate in a large multi‐ethnic cohort of patients while identifying certain marginalised patient populations, such as those with viral liver disease, cirrhosis, or African ethnicity, as particularly vulnerable to loss to follow‐up.

**Summary**

- Using an automated recall system, we were able to develop a programme capable of demonstrating a strong retention rate for patients appropriate for hepatocellular carcinoma screening, with 60% of patients retained in the programme.
- Patients were more likely to drop out if they were of African ethnicity, had Hepatitis C disease of any severity, or Hepatitis B with known cirrhosis.
- However, all groups demonstrated decreased adherence over course of the study—suggesting adherence to surveillance programmes is still an area needing further research and innovation.

## 1 Introduction

Hepatocellular carcinoma (HCC) is the third leading cause of cancer‐related death worldwide [1, 2, 3]. As per international guidelines, HCC surveillance is recommended in high‐risk patients, with biannual abdominal ultrasounds and alpha‐fetoprotein measurement [4, 5, 6]. These recommendations are based on evidence that diagnosis of early HCC improves median survival, even exceeding 5 years due to early tumour detection and application of curative treatment [7]. Unfortunately, most HCC patients are diagnosed at an advanced stage, when treatment options are limited, leading to a median survival of only 3–6 months [8]. This poor survival rate may be due to low surveillance rates of less than 24% reported to date [9, 10].

Surveillance programmes are complex, and there are multiple potential system, provider, and patient‐related factors contributing to incomplete adherence rates to surveillance [11]. In two studies, physician failure to order HCC surveillance was the most common reason, while another study examining physician practices found only 22% relied on biannual imaging for HCC surveillance [12, 13, 14]. Patient‐related characteristics driving non‐adherence are related to feasibility (cost, insurance coverage, difficulty navigating system, transportation), patient perceptions (knowledge regarding the need for surveillance, HCC presentation, management), race, and sex [10, 15, 16, 17, 18].

Several strategies, with variable success, have been explored to improve HCC surveillance adherence, including education of primary care providers, nurse‐led clinics, patient outreach by mail‐out, diagnostic imaging (DI)‐led programmes, and EMR‐led best practice alerts [19, 20, 21, 22, 23]. Care delivery changes may be associated with higher rates of surveillance, although they presumably would lead to higher costs. For example, in Australia, 53% of patients received appropriate ultrasounds following the introduction of a nurse‐led clinic. In comparison, a more intense system redesign and patient education programme led to 63% of patients being screened [24, 25] Although individual centre and region‐based initiatives have been assessed, there is limited evaluation for broader systematic programmes that can be sustained over time. The objective of the current study was to describe the impact of a city‐wide automated recall programme on HCC surveillance adherence in a cohort of high‐risk patients while defining risk factors for non‐retention in the programme.

## 2 Methods

### 2.1 Study Population

This was a retrospective study of patients who underwent HCC surveillance using abdominal ultrasound (US) through the Calgary Liver Unit (CLU) in Calgary, Alberta, Canada (~1.6 million) between January 1, 2013, and December 31, 2022. In 2013, the CLU partnered with a DI provider in Calgary (EFW Radiology, www.efwrad.com) to create an automated protocol‐based surveillance programme, based on the software used for a provincial breast cancer surveillance programme using mammography, for patients eligible for HCC screening. Patients with a confirmed diagnosis of cirrhosis (defined from a combination of biochemical investigations, radiology, pathology, and endoscopy) of any aetiology were eligible for surveillance. Non‐cirrhotic patients with chronic hepatitis B (CHB) infection were also offered surveillance if they met guideline recommendations for CHB surveillance [4, 5, 6]. Patients with a prior history of HCC, or liver transplantation, or with significant co‐morbidities that would not be eligible for HCC treatment were excluded. Patients were enrolled in this programme by any healthcare provider (primary care, gastroenterology, hepatology) by submitting a completed one‐page requisition with demographics including race, indication for screening, family history of HCC, and aetiology of liver disease/cirrhosis if applicable (Table S1). Other data collected included patient demographics, cirrhosis aetiology, total period on surveillance and total number of US performed. Once enrolled in the programme, patients underwent a complete abdominal US that was interpreted by an expert radiologist who had completed additional training in abdominal US imaging. This was utilised to place patients in a specific protocolised manner dependent on a pre‐defined HCCRAD score which was developed locally (Table S2). This placed the patient in appropriate automated recall according to the risk of HCC and was booked accordingly for ongoing surveillance imaging by the administrative team. Imaging was completed at locations suitable for the patient. This step was carried out each time, with imaging forwarded to the DI team for review as per the pre‐defined HCCRAD score. Thus, this limited the need for hepatologist appointments to review imaging after each scan. However, if the patient was flagged based on HCC RAD as high risk, an appointment was then initiated to assess for further investigation.

If patients were not able to be contacted by the DI team for ongoing surveillance US, they were attempted to be contacted one further time, thus two total attempts, in the patient's native language, as applicable. In addition, a letter to the referring physician was sent to inform them the patient was not booked for surveillance, and for the patient to call the clinic to re‐book a surveillance US. In the case of recent refugees where a dialect was not spoken by the DI provider, a letter with the relevant information in place of the US was sent to the clinic where the patient received care, often a specialised newcomer health clinic. Patients who were not able to be booked were given a 1‐year grace period before they were required to ‘re‐enrol’ into the programme. Both patient and ordering physician received a copy of the US report that listed instructions for the timing of the next follow‐up US (i.e., 3 or 6 months later) or further investigations based on their evaluation (ex. another surveillance imaging modality such as CT or MRI). Patient reports were available in English, Cantonese, Mandarin, and Vietnamese. Crucial to study outcomes, if a patient was classified as HCCRAD 3, this meant they had a high‐risk lesion identified on ultrasound > 1 cm, and these patients were considered as adherent in our primary analysis.

### 2.2 Data Acquisition, Definitions and Endpoints

Regarding demographic data collected, cirrhosis was defined based on provider judgement on initial checklist form on referral. The date of the first surveillance US served as the index date. Time followed was identified as time from first US to date of last status, be it last scan, date determined as dropped out for medical reasons, or date determined as lost in follow‐up. The number of subsequent scans and their interval dates were recorded. Complete follow‐up was defined as the patient receiving: (1) imaging every six months until either the end of the study, (2) when HCC was suspected on surveillance and subsequently confirmed on contrast imaging or with biopsy, or (3) if the referring clinical team requested cessation of surveillance. Patients with inconsistent surveillance, defined as receiving at least one scan, but without appropriate follow up imaging, were considered not retained for this study and deemed lost to follow‐up. Acceptable surveillance was defined as at least one US within one year, with no scans within 1 year of last scan deemed a drop‐out from programme. This was done based on evidence of similar studies employing an annual model when assessing the efficacy of individual surveillance programmes [26, 27, 28, 29, 30]. Although this is extended beyond society recommendations for biannual scan, this enabled us to capture patients who may have missed a scheduled 6‐month scan but were able to follow‐up within a year to ensure they were involved in surveillance practices. This is also in keeping with our programmes policies for a 1‐year grace period post‐scan before a patient would be required to contact the programme to be re‐incorporated into surveillance practices.

### 2.3 Statistical Analysis

Patient characteristics were described as counts with proportions for categorical data or medians with interquartile ranges (IQR) for continuous data. Differences in proportion were assessed using Fisher's exact test. For continuous data, Kruskal‐Wallis or Mann–Whitney U tests were used as appropriate. Cox regression models were used to identify independent non‐retention predictors compared to patients who were still enrolled or left for medical reasons. In our models, we evaluated sex, age at enrolment, ethnicity, and aetiology as independent predictors. Kaplan‐Meier analysis was used to evaluate the effect of each of our predictors on retention. The hazard ratio for each predictor was used to determine whether the variable contributed to the likelihood of not being retained. We also conducted a sensitivity analysis limiting our study between January 2013 and March 2020 to evaluate retention rates before the COVID‐19 pandemic. Further, an interaction analysis was completed which included patients with and without cirrhosis, including those with unknown aetiology that were not included in main analysis. An alpha value of 0.05 was used to assess statistical significance. This cohort study was approved by the institutional review boards at the University of Calgary (HREBA.CC‐16‐0626). Given the retrospective nature of the study, a waiver of informed consent was granted by the institutional review board. We followed the Strengthening the Reporting of Observational Studies in Epidemiology (STROBE) reporting guideline for cohort studies (Table S3).

## 3 Results

### 3.1 Surveillance Cohort

A total of 8796 patients were included in the study cohort between January 1, 2013, and December 31, 2022. Patients incorrectly entered into surveillance (*n* = 3) and those with no aetiology or incomplete data were excluded (*n* = 1528). Thus, 7269 patients were eligible for analysis. The median age at enrolment was 55.5 years (IQR: 45.5–63.8), 4355 (60%) were male and 3149 (46%) were of Asian descent. The most common aetiology for surveillance was HBV, *n* = 3734 (51%), followed by alcohol, *n* = 1085 (15%), HCV, *n* = 842 (12%), MASLD, *n* = 624 (9%), and autoimmune/genetic, *n* = 302 (4%). There were 2658 (37%) with cirrhosis with the most common aetiology being alcohol (35%), followed by MASLD (17%) and HCV (15%), HBV (12%), Other (7%) and autoimmune (6%). Median follow‐up was 1.89 years (IQR: 1.0–4.8) and the median rate of US/year was 1.82 (IQR: 1.15–2.08) during the study period. 113 (1.6%) of patients died during follow‐up (with reasons not available). The average year‐over‐year loss to follow‐up rate was 7%. Data on the referring medical specialist (i.e., hepatology, gastroenterology, primary care, etc.) was not available. Characteristics of the study cohort are outlined in Table 1.

**TABLE 1:** Patient demographics of HCC surveillance cohort.

Variable	Overall cohort	Retained	Left for medical reason^b^	Not retained	*p*
	7269	3768	603	2898	
Male (%)	4355 (59.9)	2172 (57.6)	411 (68.2)	1772 (61.1)	< 0.01
Age at enrolment (years) Median, IQR	55.51 [45.46, 63.77]	55.41 [45.81, 64.05]	59.57 [50.48, 67.06]	54.73 [44.02, 62.79]	< 0.01
Years in study, Median, IQR	1.89 (1.00–4.08)	2.71 (1.00–5.57)	1.00 (1.00–1.42)	1.58 (1.00–2.82)	< 0.01
Scans/year, Mean (SD)	1.82 (1.15–2.08)	2.02 (1.92–2.23)	2.00 (1.00, 2.64)	1.33 (1.00, 1.58)	< 0.01
Deceased (%)	113 (1.6)	0 (0.0)	113 (17.8)	0 (0.0)	< 0.01
Primary Center of residence (Calgary) (%)	6549 (90.2)	3442 (91.3)	519 (86.4)	2588 (89.5)	0.01
Ethnicity *n* (%)					
Asian	3147 (46.4)	1873 (54.4)	164 (28.9)	1110 (40.1)	< 0.01
Caucasian	2511 (37.0)	1071 (31.1)	324 (57.1)	2511 (37.0)	
African	647 (9.5)	302 (8.8)	31 (5.5)	314 (11.3)	
Other/Unknown	474 (7.0)	198 (5.7)	48 (8.5)	228 (8.2)	
Aetiology					
HBV	3734 (51.4)	2205 (58.5)	181 (30.0)	1348 (46.5)	< 0.01
HCV	842 (11.6)	253 (6.7)	65 (10.8)	524	
Alcohol	1085 (14.9)	511 (13.6)	125 (20.7)	449 (15.5)	
MASLD	624 (8.6)	352 (9.3)	98 (16.3)	174 (6.0)	
Autoimmune/Genetic^a^	302 (4.2)	118 (3.1)	40 (6.6)	144 (5.0)	
Other	282 (3.9)	138 (3.7)	51 (8.5)	93 (3.2)	
Multiple aetiologies	400 (5.5)	191 (5.1)	43 (7.1)	166 (5.7)	
Cirrhosis (by Aetiology) *n* (%)					
Overall	2658 (36.6)	1338 (30.8)	377 (59.3)	943 (41.2)	
HBV	328 (12.3)	124 (10.8)	36 (10.1)	168 (14.6)	< 0.01
HCV	399 (15.0)	107 (9.3)	35 (9.8)	257 (22.3)	
Alcohol	923 (34.7)	417 (36.4)	116 (32.5)	390 (33.8)	
MASH	439 (16.5)	249 (21.7)	72 (20.2)	118 (10.2)	
Autoimmune/Genetic	145 (5.5)	59 (5.1)	27 (7.6)	59 (5.1)	
Multiple aetiologies of disease	229 (8.6)	116 (8.7)	33 (8.8)	80 (8.5)	
Other	195 (7.3)	91 (7.9)	40 (11.2)	64 (5.5)	
Family history HCC *n* (%)	322 (6.5)	183 (9.7)	19 (3.7)	120 (4.7)	< 0.01
HCC rads score *n* (%)					
0	428 (5.9)	0 (0.0)	428 (71.0)	0 (0.0)	< 0.01
1	6033 (83.0)	3546 (94.1)	167 (27.7)	2320 (80.1)	
2	193 (2.7)	81 (2.1)	4 (0.7)	108 (3.7)	
3	615 (8.5)	141 (3.7)	4 (0.7)	470 (16.2)	

^a^ Autoimmune/Genetic includes Autoimmune hepatitis, Primary biliary cholangitis, Primary sclerosing cholangitis, Alpha‐1 antitrypsin deficiency, Wilson's disease, and Hemochromatosis.

^b^ Left for Medical Reasons meant death or having a lesion that required further contrast‐enhanced imaging HCC.

### 3.2 Surveillance Programme and Predictors of Retention

Overall, 3768 (51.8%) of patients were retained in the surveillance programme, while 603 (8.3%) left for potential medical reasons during the study period, thus, leaving 2898 (40%) patients not being retained. Of the patients leaving for medical reasons, 110 (18%) died, with further medical data not available. The median time in the programme for those lost in follow‐up was 1.58 years (IQR: 1.00–2.82) versus 2.71 years (IQR: 1.00–5.57; *p* < 0.001) for those who were retained. The number of scans was also highest in retained cohort, with median 2.02 scans/year (IQR 1.00–2.64) versus median 1.33 (IQR 1.00–1.58) scans in patients lost in follow‐up. Subjects lost in follow‐up were more likely to be male (61% vs. 58%; *p* < 0.001), and White (40% vs. 31% in retained patients). Compared to patients lost to follow‐up, those retained were more likely to be Asian (54% vs. 40%, *p* < 0.01). Regarding the aetiology of liver disease, patients with HCV (18% vs. 11%; *p* < 0.01) were significantly more represented in the lost to follow‐up cohort.

**FIGURE 1:**
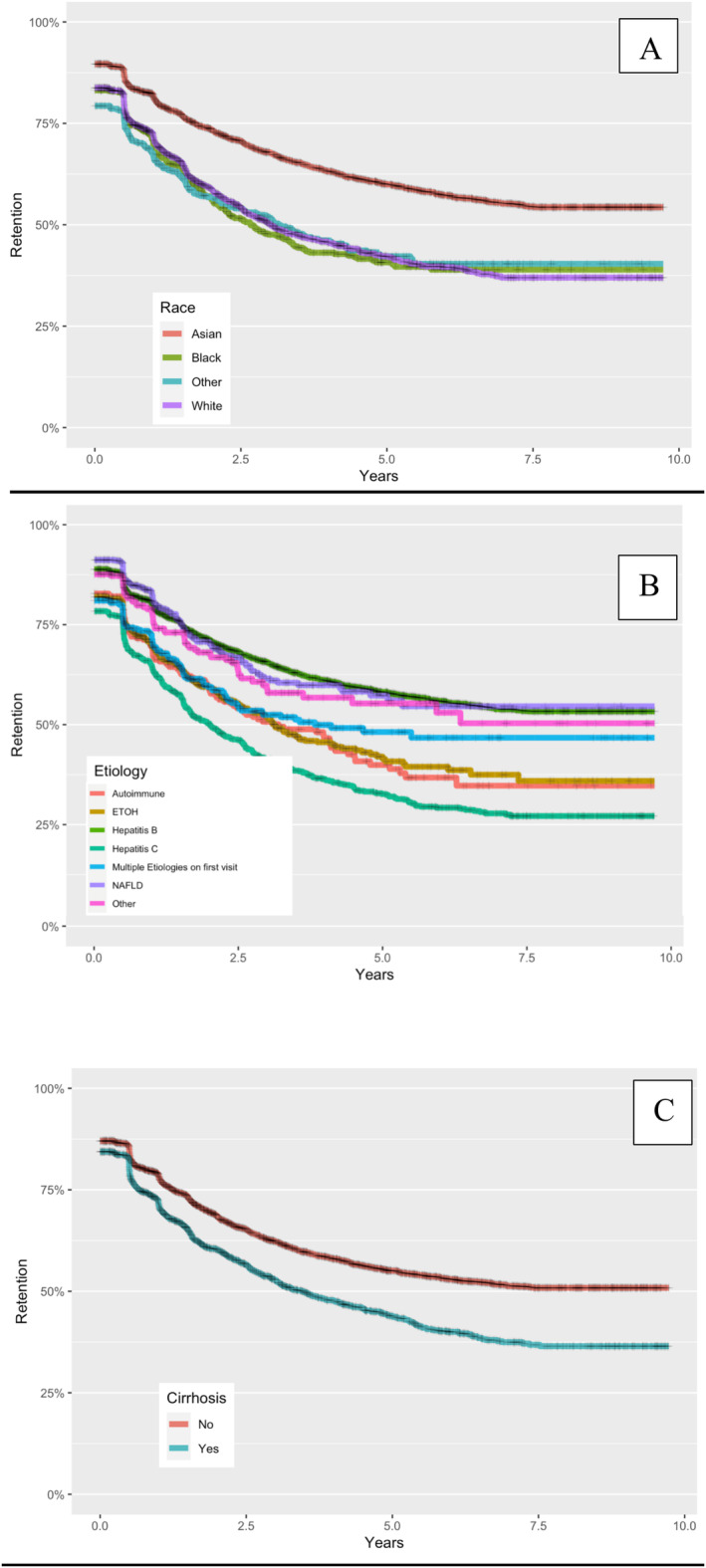
HCC surveillance rates stratified by (A) race (B) aetiology and (C) presence of cirrhosis.

Although globally there was a decrease in surveillance rates over time, we found rates of surveillance were lowest over time for those with HCV infection, of white race, and patients with cirrhosis over the study period amongst their respective cohorts (Figure 1). For HCV patients, the loss‐to‐follow‐up rate in the first year was 27.5%, and in the fifth year was 6.4%, with the cumulative loss‐to‐follow‐up rate of 50.63%. For cirrhosis patients, the loss‐to‐follow‐up rate in the first year was 20.2%, and in the fifth year was 3.8%, with the cumulative loss‐to‐follow‐up rate of 34.1%. For white patients, the loss‐to‐follow‐up rate in the first year was 20.9%, and in the fifth year was 4.0%, with the cumulative loss‐to‐follow‐up rate of 36.3%. For reference, the initial frequency of loss to follow‐up for patients in year 1 was 13.5%, the rate in 5th year of follow‐up was 2.4%, and overall, the rate over 5 years was 24.4%.

A subgroup analysis was also done of patients who were involved in surveillance during COVID‐19 pandemic to assess loss to follow‐up. The year‐by‐year lost to follow‐up rate immediately pre‐pandemic in 2019 was 18.75; however, the rate in 2020 was 16.51. The drop‐out rate for patients included beyond is not available as they did not meet the 2‐year cutoff during follow‐up to be considered out of the programme.

### 3.3 Multivariable Analysis

In multivariable Cox regression analysis, HCV infection (HR 1.41; CI 1.23–1.62, *p* < 0.01), African ethnicity (HR 1.20, CI 1.02–1.42, *p* = 0.03), and cirrhosis (HR 1.16, CI 1.05–1.28, *p* < 0.01) were predictors of loss to follow‐up. In comparison, older age (HR 0.99, CI 0.99–0.99, *p* < 0.01), Asian ethnicity (HR = 0.76; CI 0.68–0.87; *p* < 0.01) and aetiologies of HBV (HR = 0.75; CI 0.64–0.88; *p* < 0.01) and MASLD disease (HR = 0.69; CI 0.58–0.83; *p* = 0.01) were identified as predictors of reduced dropout rate. No other aetiology of liver disease was significant in multivariable analysis, including patients comorbid with multiple aetiologies of liver disease. Data is summarised in Table 2 for further review. An interaction analysis was completed following this. Aetiology of disease on interaction analysis was mildly significant between age of enrolment and cirrhosis (HR 1.01, CI 1.00–1.02, *p* < 0.01), and HCV (HR 1.46, CI 1.08–1.97, *p* < 0.01). Although Hepatitis B itself was a predictor of better adherence, patients with Hepatitis B and cirrhosis were at greater risk of loss in follow‐up (HR 1.48, CI 1.05–2.07, *p* = 0.02) (Table S4).

**TABLE 2:** Results of multivariable cox regression model predicting patients not retained in the HCC surveillance programme.

Variable	Univariable HR	95% CI	Multivariable HR	95% CI	*p*
Age	0.99	0.99–1.00	0.99	0.98–0.99	< 0.01
Gender (male)	1.11	1.03–1.19	—	—	—
Race (White)					
Asian	0.60	0.55–0.65	0.76	0.68–0.87	< 0.01
Black	1.04	0.92–1.18	1.20	1.02–1.42	0.03
Other	1.04	0.90–1.20	1.11	0.95–1.28	0.18
Aetiology (alcohol)					
HBV	0.62	0.56–0.70	0.76	0.64–0.88	< 0.01
HCV	1.28	1.13–1.46	1.41	1.23–1.62	< 0.01
Autoimmune	1.02	0.85–1.23	1.13	0.93–1.37	0.24
MASLD	0.62	0.52–0.74	0.69	0.58–0.83	< 0.01
Multiple aetiologies	0.92	0.77–1.10	0.96	0.79–1.16	0.65
Other	0.71	0.57–0.89	0.75	0.60–0.95	0.02
Diagnosed cirrhosis (yes)	1.38	1.28–1.48	1.16	1.05–1.38	< 0.01

*Note:* Reference group in parenthesis for each cohort.

## 4 Discussion

In this large and racially diverse population‐based study, we show that a dedicated automated HCC surveillance programme can achieve a high (60%) and sustainable retention rate. Further, significant predictors of non‐retention in our programme were identified which included HCV infection and presence of HBV‐associated cirrhosis.

There are multiple factors impacting the success of traditional HCC surveillance. These barriers include provider limitations, such as not feeling up to date with surveillance guidelines and not being able to identify cirrhosis patients [16]. Importantly, patients may face financial barriers (travel requirements, time off work, childcare, etc.) as well as cultural and language barriers, while having to navigate a complex medical system fraught with issues of capacity, facility shortages, and challenges with recall [10]. We have shown that a multidisciplinary partnership with a DI provider can mitigate a number of these system‐related issues. Moreover, this study demonstrates that only a small percentage of patients are not retained when enrolled in a rigorous recall system. Identification of non‐retention occurs early during follow‐up, given significant proportion of drop‐off occurs within a year after enrolment in the programme. Although we identified a cohort of patients historically recognised as being more likely to be non‐compliant/adherent due to sociodemographic factors, we have shown that after 5 years of surveillance retention rates decline for all groups, irrespective of race or aetiology of liver disease, including cirrhosis (Figure 1). Thus, patients are not meeting best practices of having biannual US, as the average US in our retained cohort was 1.2 per year [31]. Although not addressed in this study, one possible explanation for this drop‐off could be patient fatigue. Specifically, if a patient lacks symptoms and has a normal physical exam, or they do not understand why they require ongoing screening/testing beyond a certain time point they may be less likely to remain enrolled [32, 33, 34]. The COVID‐19 pandemic also likely impacted surveillance retention in the years 2020–2022. Although we do not have the comprehensive 2023 and 2024 data for these patients in our database, this should also be considered moving forward.

Our findings add to the dialogue around how best to refine surveillance programmes for HCC to improve patient retention [35]. Recently, the European Association for the Study of the Liver (EASL) stated in a consensus document that up to 20% of patients considered low risk for HCC can avoid screening, while higher‐risk individuals should be offered more intensive follow‐up surveillance (i.e., abbreviated MRI) [36]. Liver disease as a risk factor for HCC would be ideal for such a strategy, as previous literature has shown a reduced risk of HCC in certain liver disease aetiologies, and an increased risk in other aetiologies like CHB [36, 37]. That viral disease was a factor increasing risk of drop‐out in both cirrhotic and non‐cirrhotic cohorts further elucidates a known barrier regarding viral hepatitis populations and linkage to both treatment and screening—often exacerbated in marginalised populations [38]. We were able to also identify that the African Canadian population is at further risk of increased loss to follow‐up, concerning given there are multiple studies identifying multi‐modal barriers to care for this population. Identifying such populations is crucial for refining our existing models of surveillance to further target high‐risk patients, while deferring screening in low‐risk cohorts, mitigating the harms of surveillance (i.e., anxiety, unnecessary tests) while being cost‐effective [36, 37, 39, 40].

Several reviews summarising the effectiveness of interventions promoting adherence to surveillance programmes have revealed that only roughly half of the interventions were associated with a significant increase in adherence, and even fewer reported an improvement in treatment outcomes [19, 20, 21, 41]. In large‐scale community‐based surveillance programmes, as done in the current study, there are a few core principles that can be applied to any screening programme to enhance appropriateness and adherence. First, we established clear criteria for appropriate patients for surveillance, outlining which liver disease patients were eligible for screening and using a checklist to develop a consistent workflow. Second, we defined the frequency of the intervention before initiating the programme, surveillance being every 6 months in this case, with standardised reporting (HCCRAD score) to guide users if enhanced surveillance (every 3 months) or if contrast imaging (CT, contrast‐enhanced ultrasound, MRI) tests were required. This feedback system also included instructions regarding when a primary care provider should refer their patients to a hepatologist (> 1 cm lesion). Third, we also included an automatic follow‐up system in our medical reporting system (‘your patient will be re‐booked for the US in 6 months’) so practitioners would know who was responsible for organising the next screening dates, and that their patient was still enrolled. Lastly, the success of community‐based screening programmes, such as this one, requires close engagement with community and patient partners, including the creation of educational and culturally relevant educational materials, for example, handouts in several languages, as well as providing a copy of the US result to the patient in their native language.

The strength of this study is that it included a large racially diverse, at‐risk patient population with various liver disease aetiologies that was established in a single‐payer, universal healthcare access setting. This enables us to identify demographics‐driven differences in population retention that apply to other centres. However, there were limitations. First, there are inherent limitations given this is a retrospective monocentric study, with no comparative cohort as a control. We also recognise the inherent limitations of the database, given these are patients pre‐referred by providers, granular data regarding liver disease characteristics, such as HBV viral load, response to viral therapy, BMI, comorbidities, degree of portal hypertension, incidence of HCC, treatment of HCC and overall survival were not included. Further, specific indications for why patients were removed for medical reasons, specific details regarding diagnosis of cirrhosis, as well as the specialty of provider, were not collected. Further, we recognise the HCCRAD report is not validated, but mimics the currently used American College of Radiology (ACR) ultrasound screening and surveillance algorithm and was instituted in an attempt to make the requisition and reporting forms standardised, easy to use and interpret, including a safety net advising to the consideration of early referral to Hepatology if a concerning lesion (i.e., HCCRAD 3) was identified [42]. This methodology also does not include AFP, and we recognise this does not reflect ideal surveillance protocols. Lastly, we acknowledge the limitations of our annual cutoff. While there is utility for this cutoff from a practical standpoint to include patients who may be slightly beyond the 6‐month interval, we recognise this is a limitation as it does not fit within standard of care. There is varied evidence from prior programmes on how to best evaluate adherence to surveillance programmes, with studies demonstrating a split between either detection by an HCC surveillance modality or some form of cutoff, ranging from 6 months to 36 months between scan [43]. A one‐year cutoff is used in multiple studies, and clearly is an improvement in existing trends, with some studies reporting more than 1/3 of new HCC diagnoses were associated with no imaging or investigation in year prior to diagnoses [43]. The intention was to identify a threshold at which patients can be considered as dropped out from surveillance with a high degree of confidence and thus set accordingly.

## 5 Conclusions

In this large and racially diverse cohort of patients eligible for HCC surveillance enrolled in a dedicated automated recall system, we found retention rates. In addition, we identified several modifiable and non‐modifiable risk factors for non‐retention. Future studies are being planned to evaluate the long‐term outcomes and resource utilisation of this programme.

## Author Contributions

Authors had access to all the study data and took responsibility for the accuracy of the analysis and had final authority over manuscript preparation and the decision to submit the manuscript for publication. All authors approve the manuscript.

## Ethics Statement

Ethics approval was received from the institutional review boards at the University of Calgary.

## Consent

Waiver of informed consent was granted by the institutional review board.

## Conflicts of Interest

Mark Swain: Advisory Board: Ipsen, Pfizer, Advanz, Novo Nordisk, GSK; Speaker: Gilead, Abbott; Clinical trial or research support: Gilead, BMS, CymaBay, Intercept, Genfit, Pfizer, Novartis, Astra Zeneca, GSK, Celgene, Novo Nordisk, Axcella Health Inc., Merck, Galectin Therapeutics, Calliditas Therapeutics, Madrigal, AbbVie, Altimmune, Roche, Kowa.

## Supporting information


Data S1.

## Data Availability

Research data are not shared.
